# Firefighter cancer risks: a systematic review and proposal for a volunteer-specific decontamination model

**DOI:** 10.1093/joccuh/uiag013

**Published:** 2026-03-07

**Authors:** Ben Schutte, Oluwabunmi Dada, Rachael Obeng, Traci Byrd

**Affiliations:** Department of Occupational Safety and Health, Murray State University, 157 Industry and Technology Center, Murray, KY 42071, United States; Calloway County Fire Rescue, Murray, KY 42071, United States; Department of Occupational Safety and Health, Murray State University, 157 Industry and Technology Center, Murray, KY 42071, United States; Department of Occupational Safety and Health, Murray State University, 157 Industry and Technology Center, Murray, KY 42071, United States; Department of Occupational Safety and Health, Murray State University, 157 Industry and Technology Center, Murray, KY 42071, United States

**Keywords:** firefighters, cancer risk, cancer prevention, PPE, decontamination models, occupational health

## Abstract

**Objectives:**

Firefighters face a disproportionately high risk of developing cancer due to occupational exposure to carcinogens, including combustion byproducts, diesel exhaust, and per- and polyfluoroalkyl substances (PFAS) found in firefighting foams and protective gear. This study aimed to systematically review existing literature on cancer risks among firefighters, with particular attention to exposures, prevention strategies, and decontamination practices, and to propose a novel model addressing current gaps.

**Methods:**

A systematic review was conducted in accordance with the Preferred Reporting Items for Systematic Reviews and Meta-Analyses guidelines. Peer-reviewed articles, international reports, and high-quality training materials were included. A total of 37 records were included in the final review. Data were synthesized across thematic areas relevant to firefighter cancer risk.

**Results:**

Five key themes were identified: (1) cancer incidence and mortality, (2) occupational exposure sources, (3) basic prevention practices, (4) gear hygiene, and (5) station decontamination models. The review also identified a critical gap in current decontamination protocols for volunteer firefighters in rural areas, who often respond to emergencies using personal vehicles. To address this, we proposed a novel, volunteer-specific decontamination framework, the Schutte Model, adapting existing best practices to minimize carcinogen transfer during gear transport.

**Conclusions:**

This study provides both an evidence-based synthesis and a novel intervention model. Findings underscore the urgent need for inclusive, low-cost, and culturally tailored prevention strategies to reduce cancer risks among all firefighters, particularly volunteers operating in under-resourced and decentralized contexts.

## Teaser

**Key points**

**What is already known on this topic**: Firefighters are at an elevated risk of developing several cancers, including testicular cancer, non-Hodgkin lymphoma, bladder cancer, mesothelioma, and melanoma. Occupational exposures such as combustion byproducts, diesel exhaust, and per- and polyfluoroalkyl substances (PFAS) from firefighting foams and protective gear are recognized contributors to these risks. Existing decontamination and prevention models, such as the Skellefteå model, have been developed primarily for career firefighters working from centralized fire stations.

**What this study adds**: This systematic review, conducted following PRISMA guidelines, consolidates international literature, reports, and training materials into 5 thematic areas: cancer incidence and mortality, occupational exposures, prevention practices, gear hygiene, and station decontamination models. A critical gap was identified in protocols for volunteer firefighters, particularly in rural areas where personal vehicles are used for emergency response. To address this gap, the study proposes the Schutte Model, a novel, low-cost decontamination framework adapted from existing best practices, explicitly designed to reduce carcinogen transfer in volunteer firefighter contexts.

**How this study might affect research, practice, or policy**: The findings reinforce the need for inclusive occupational cancer prevention strategies that extend to volunteer and under-resourced fire departments. Adoption of the Schutte Model and similar low-cost interventions could inform organizational policies, improve gear-handling protocols, and protect firefighters’ families from secondary exposures. Policymakers and occupational health practitioners can use this evidence to support culturally tailored cancer prevention initiatives, funding mechanisms, and regulatory updates that reflect the diverse realities of firefighting.

## 1. Introduction

Firefighters routinely face exposure to hazardous environments and chemical contaminants during fire suppression, rescue operations, and post-fire cleanups.^1^ These occupational exposures have been linked to a disproportionately high incidence of cancer among firefighters compared with the general population.^2–4^ Previous studies showed elevated risks for different types of cancers, including but not limited to, multiple myeloma, non-Hodgkin lymphoma, colon, bladder, and testicular cancer, and mesothelioma, among others.^3–6^ The carcinogenic exposures stem from a range of sources, including combustion byproducts from modern building materials,^7^ diesel-powered fire engines creating diesel engine exhaust (DEE) particles,^8,9^ per- and polyfluoroalkyl substances (PFAS) in firefighting gear and foams, and contaminated personal protective equipment (PPE).^2,10–13^

Although many of the hazardous substances encountered during fire events are not unique to firefighters, the nature of firefighting work results in a distinct exposure profile characterized by repeated, high-intensity exposures to complex mixtures of carcinogens under extreme thermal and physical conditions.^14^ Firefighters experience simultaneous inhalation, dermal, and secondary exposure pathways, including prolonged contact with contaminated PPE during overhaul, transport, and storage.^11^ These cumulative and occupation-specific exposure patterns differentiate firefighters from other occupations that may encounter similar substances in more controlled or intermittent settings.

The diversity in firefighter roles across jurisdictions adds further complexity to cancer risk assessments.^11^ In the United States more than 65% of the firefighting workforce is composed of volunteers.^15^ A study by Tonnaer^16^ in 2019 summarized the legal status of volunteer firefighters in Europe, highlighting how countries like Germany, Austria, Latvia, and Estonia mainly rely on volunteer firefighters. Unfortunately, these individuals often lack the institutional resources and standardized decontamination protocols available to career firefighters.^17^ A critical distinction lies in how they respond to emergencies: whereas career firefighters deploy from central stations using fire apparatus, volunteers, especially in rural settings, frequently store their turnout gear at home and respond to incidents in personally owned vehicles (POVs).^18^ This decentralized response model increases the likelihood of prolonged contact with contaminated gear, raising exposure risks for both firefighters and their families.^19,20^ Furthermore, since most volunteer firefighters typically have other full-time jobs, this could mean they will spend less time training than career firefighters.^21^

Despite a growing body of research on firefighter cancer risks, volunteer firefighters remain vastly underrepresented. For example, major epidemiological studies have focused exclusively on career personnel, overlooking the 65% of the US firefighting workforce that serves on a volunteer basis.^2,4^ This gap limits understanding of their unique exposure risks, especially given their dual occupations and lack of access to station-based decontamination protocols.^22^ Additionally, most prevention models are designed for career departments, with few addressing rural or POV-based response. The literature remains fragmented, often focused on single cancer-related topics,^8,23,24^ and rarely includes emerging risks such as PFAS-contaminated bunker gear.^25–27^ Cancer outcomes were selected as the primary focus of this review because cancer represents the leading cause of line-of-duty death among firefighters and reflects the long-term health consequences of cumulative occupational exposure,^5,11,28^ distinguishing it from acute or short-term health effects.

The distinction between career and volunteer firefighters is not introduced to facilitate a direct comparison of cancer incidence between these groups, but rather to highlight a critical prevention and implementation gap in the existing literature. While much of the epidemiological evidence on firefighter cancer risk is derived from career cohorts,^2,4^ the majority of firefighters globally serve in volunteer or mixed-response systems,^15,16^ where infrastructure, training, and access to decontamination resources differ substantially. These structural differences have direct implications for exposure persistence and secondary contamination pathways, particularly for firefighters responding from POVs.

Different factors contribute to elevated cancer rates among firefighters, including insufficient awareness, limited training, and the absence of standardized preventive policies.^5,19,29,30^ Cultural attitudes and behavioral norms, as highlighted in the works of Wolffe et al,^6,30,31^ further complicate risk mitigation, often discouraging decontamination and proper PPE use. Although no intervention can eliminate exposure entirely, consistent application of best practices and modern PPE can significantly reduce risk.^1^ To address gaps in protection for those responding in POVs, including volunteers and fire officers, this study proposes a new decontamination model adapted from existing frameworks like the Skellefteå model,^29^ tailored to the needs of decentralized responders.

A review organized solely around hazardous substances encountered at fire scenes would not adequately capture the occupational context in which firefighters are exposed, including work organization, response models, PPE practices, decontamination behaviors, and cultural norms that shape exposure duration and magnitude. Building on this foundation, this study does not aim to quantitatively compare cancer risks between career and volunteer firefighters, but rather to systematically synthesize evidence on occupational cancer-related exposures, cancer incidence and mortality, and prevention strategies among firefighters, while explicitly considering how decentralized and resource-limited response models shape exposure risk. Using this evidence base, the study further seeks to inform the development of a practical, low-cost decontamination framework tailored to firefighters operating in decentralized response contexts, particularly volunteer firefighters responding from POVs. By integrating evidence synthesis with a context-specific intervention proposal, we address both scientific and practical gaps in current firefighter occupational health research.

## 2. Methods

This study followed the Preferred Reporting Items for Systematic Reviews and Meta-Analyses (PRISMA)^32^ guidelines to conduct a comprehensive review of firefighter cancer risks.

### 2.1. Search strategy

From August 2024 to November 2024, the investigators systematically searched peer-reviewed literature, reports, and training materials across PubMed, Scopus, Embase, and Google Scholar. Additional gray literature was identified through bibliographic screening and manual searches of government organizations and reputable firefighting training institutes, such as the University of Missouri Fire and Rescue Training Institute or New Zealand’s emergency service body. Search terms utilized were combinations of “firefighter” (1), “cancer” (2), “firefighting foam” or “Aqueous Film-Forming Foam or AFFF” or “Fluorine-Free Foam or F3 foam” (3), “diesel exhaust” or “Diesel Engine Exhaust or DEE” (4), “Gear” or “Turnout gear” or “bunker gear” (5), “Per- and Polyfluoroalkyl Substances or PFAS” (6), “mortality” (7), “incidence” (8), “Clean Gear” (9), “Personal Protective Equipment or PPE” (10), “building materials” (11), “decontamination” (12), “fluorine-free” (13), “exposure” (14). Both full terms and acronyms (eg, AFF, PFAS, etc) were used to ensure comprehensive retrieval.

This is an example of the search string used in PubMed: (“firefighter”) AND (“cancer”) AND (“mortality” OR “incidence”) AND (“firefighting foam” OR “Aqueous Film-Forming Foam” OR “AFFF” OR “Fluorine-Free Foam” OR “F3 foam”) AND (“diesel exhaust” OR “Diesel Engine Exhaust” OR “DEE”) AND (“gear” OR “turnout gear” OR “bunker gear”) AND (“Per- and Polyfluoroalkyl Substances” OR “PFAS”) AND (“clean gear” OR “personal protective equipment” OR “PPE”) AND (“decontamination” OR “exposure”). Searches were conducted using keyword-based Boolean combinations of predefined terms rather than controlled vocabulary. Both full terms and acronyms were applied to ensure comprehensive retrieval. Medical Subject Headings were not explicitly used.

### 2.2. Inclusion and exclusion criteria

The records comprised peer-reviewed journal articles, systematic literature reviews, training videos, and meta-analyses addressing firefighter cancer exposure and control. Exposures of interest included carcinogenic combustion materials, diesel exhaust, firefighting foams, contaminated gear, and PFAS in gear. Studies and videos were limited to English language publications only. The search covered globally published peer-reviewed articles and training videos between 2004 and 2024. Reports from international bodies such as the International Agency for Research on Cancer (IARC) were also included, particularly the IARC Monograph on occupational carcinogens in firefighting.

Gray literature, including training videos and technical guidance documents, was selected using predefined criteria emphasizing authority, relevance, and educational purpose. Eligible materials were produced or endorsed by recognized firefighting organizations, government agencies, or training institutes. All gray literature sources were appraised using the AACODS checklist.^33^

Duplicates and studies without relevant occupational exposure content were excluded. Titles and abstracts were initially screened by 1 reviewer to identify potentially eligible records. To enhance methodological rigor, 2 reviewers subsequently reviewed screening decisions and assessed all full-text articles for eligibility using the same inclusion criteria. Any discrepancies were resolved through discussion and consensus. Studies deemed ineligible were excluded. All remaining studies were thoroughly examined and reviewed.

### 2.3. Data extraction

Data were extracted from records meeting the inclusion criteria using Microsoft Excel. The data collected included author(s), publication year, country of origin, type of data (video or article), title, exposure type, preventive measures, and key findings.

### 2.4. Quality assessment

Given the heterogeneity of study designs included in this review, a structured multi-tool approach was employed to ensure methodological rigor and appropriate appraisal across evidence types. Cohort studies were assessed using the Joanna Briggs Institute (JBI) Cohort Checklist, while cross-sectional studies were evaluated with the JBI Cross-sectional Checklist. Experimental and quasi-experimental investigations were reviewed using the JBI Quasi-Experimental Checklist. These JBI tools provided a systematic framework to assess study selection, measurement reliability, exposure validity, outcome assessment, and control of confounding variables.^34^ Systematic reviews and authoritative monographs were appraised with the AMSTAR 2 tool,^35^ while narrative reviews were evaluated using the SANRA scale.^36^ Gray literature sources, including agency reports, policy documents, training manuals, and technical guidelines, were critically appraised using the AACODS checklist.^33^

Studies were retained if they met at least 60% of the tool-specific criteria, corresponding to minimum scores of 5-8 points across instruments. Detailed results of the quality appraisal are presented in Tables S1–S7.

### 2.5. Protocol registration

The review protocol was not registered in PROSPERO prior to study initiation, which is acknowledged as a limitation. However, the review followed PRISMA guidelines with a predefined search strategy, eligibility criteria, and structured quality appraisal to ensure transparency and reproducibility.

## 3. Results

### 3.1. Study selection

A total of 576 records were initially identified using the databases and keywords previously mentioned. An additional 8 articles and 4 training videos were identified through manual bibliographic searches. After removing duplicates, 386 unique records were screened for relevance. Based on title and abstract review, 132 records were selected for full-text assessment, and ultimately 37 peer-reviewed articles, including 4 training videos, met the inclusion criteria. Furthermore, authoritative reports from international health bodies were included, with priority given to the IARC Monograph on occupational carcinogens in firefighting, specifically volume 132. The Monograph provided essential classification frameworks for exposure risks. It reinforced the evidence base linking specific carcinogens, such as polycyclic aromatic hydrocarbons (PAHs), diesel exhaust, benzene, and PFAS, with elevated cancer risks among firefighters.

Following quality appraisal, studies that met the predefined threshold (at least 60% across tool-specific criteria) were retained for inclusion in the final synthesis. Application of this criterion resulted in the inclusion of methodologically adequate epidemiological studies, experimental investigations, and relevant gray literature. Detailed quality assessment outcomes for included studies are presented in Tables S1–S7.

**Figure 1:**
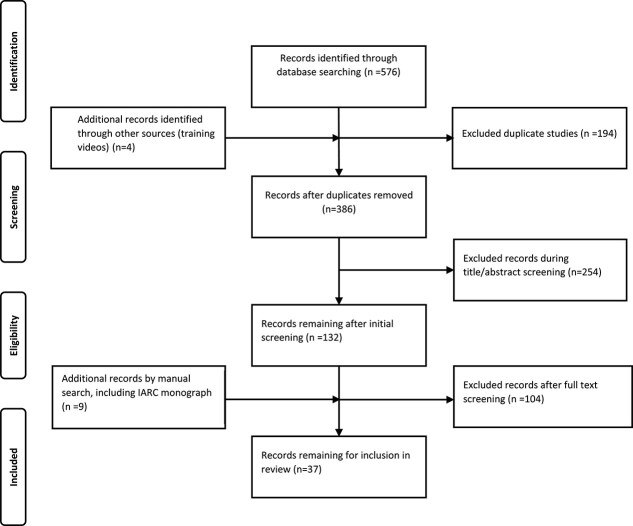
Process flow chart to identify reviewed articles.

These selected materials originated from 11 countries, with the majority published between 2014 and 2023. All materials were published in English. The PRISMA flow diagram illustrating the screening process is provided in Figure 1.

### 3.2. Study characteristics

This review included 37 records encompassing peer-reviewed articles, international reports, and training materials. Most of the studies originated from the United States, with additional contributions from Canada, the United Kingdom, Sweden, Spain, Iran, New Zealand, Taiwan, India, Slovakia, and multinational organizations such as the IARC. The majority of publications were peer-reviewed journal articles, supplemented by governmental and organizational documents that addressed firefighter health, safety, and cancer prevention practices.

Study designs varied and included large epidemiological cohort studies, exposure assessment studies, experimental evaluations of decontamination procedures, and descriptive training or policy documents. Several studies were based on national or regional firefighter cohorts, while others drew from smaller occupational groups or training institutes. The diversity of study types and geographic coverage provided a broad perspective on cancer risks, exposure pathways, and preventive strategies in the firefighting profession.

Although differences between career and volunteer firefighters are discussed in the literature, most included studies did not report stratified outcomes by employment status; therefore, results are presented at the occupational level, with implications for volunteer firefighters addressed in subsequent sections.

### 3.3. Emerging themes

To address the study aim, findings were synthesized across predefined domains encompassing cancer incidence and mortality, sources of occupational exposure, and prevention and decontamination strategies. This thematic synthesis resulted in 5 closely related topic areas relevant to cancer risk among firefighters: (1) cancer incidence and mortality, (2) sources of occupational exposure, (3) basic preventive measures, (4) effectiveness of clean PPE, and (5) station designs and decontamination models. Together, these themes provide an integrated overview of both cancer risk and mitigation evidence in firefighter occupational health. The topics discussed in each record are listed in Table S8, which also highlights the record type (article [A] or video [V]) and the corresponding quality assessment score. Of all reviewed records, 14 discussed cancer incidence and mortality, and 26 provided information on potential occupational exposure that led to the development of cancers. Twelve articles discussed preventive measures to reduce the risk of exposure. Additionally, 9 studies showed the effectiveness of clean PPE to reduce cancer exposure. Only 8 studies focused on station designs and decontamination models to prevent the travel of potential carcinogens. Several studies addressed multiple themes, resulting in overlap across these categories. The 4 reviewed training videos and reports from international bodies provided a comprehensive summary of all topics and a solid background for the study. Every reviewed study or training video mentioned the risk of developing cancer when employed as a firefighter or handling frequently used materials such as foams or contaminated fire gear.

#### 3.3.1. Cancer incidence and mortality

From the 14 articles discussing cancer incidence and mortality, all studies determined firefighters to be at a significantly elevated risk of developing some cancers compared with other occupations.^2,4,15,29,37–39^ Studies by the National Institute of Occupational Safety and Health (NIOSH) show that firefighters have a 9% higher cancer incidence and a 14% higher cancer mortality compared with the US general population.^3,4^ Six studies examined specific cancer types and reported significantly elevated risks for multiple myeloma, testicular cancer, non-Hodgkin lymphoma (NHL), bladder, colon, rectal, prostate, and thyroid cancers, as well as mesothelioma and malignant melanoma.^2–5,11,38^ Additionally, two articles identified an increased risk for firefighters of races/ethnicities other than white for non-Hodgkin lymphoma, leukemia, and tongue, testis, and bladder cancers in addition to all other previously mentioned cancers.^2,3^ The 2023 IARC Monograph Volume 132 corroborates these findings, officially classifying firefighting as a Group 1 carcinogenic occupation, citing sufficient evidence of cancer in humans due to occupational exposures.^14^ This classification provides a strong global validation of the epidemiological patterns observed in peer-reviewed literature. All articles discussing cancer incidence and mortality recommended improving technologies and education of firefighters to reduce exposure.

#### 3.3.2. Sources of occupational exposure

Twenty-six studies detailed the risk of exposure to known hazards during firefighting duties.^40–44^ These exposures occurred across a wide range of job tasks, including fire suppression, rescue operations, and hazardous materials response. Six articles discussed general occupational exposure, highlighting the risks associated with structural fires, vehicle accidents, and chemical spills.^2–5,11,45^ Within this category, 8 additional records offered more specific detail on carcinogenic combustion materials and related exposures.^10,12,13,19,20,26,38,46^

The most frequently reported carcinogens included PAHs, volatile organic compounds (VOCs), diesel exhaust particulates, formaldehyde, benzene, and PFAS.^11,12,19,25,26^ Reports from the IARC Monograph Volume 132 reinforce the identification of these substances as high-risk exposures frequently encountered during fire suppression and overhaul phases.^14^ Overhaul is the post-fire process of thoroughly searching for, locating, and extinguishing any remaining hidden fires, embers, or hot spots to avoid rekindling.^47^ For example, PAHs and DEE are listed by the IARC as Group 1 carcinogens, indicating sufficient evidence of carcinogenicity in humans. PFAS, particularly those present in firefighting foams and turnout gear, are classified as emerging risks requiring urgent mitigation.^14^

Two articles examined the exposure to DEE particles from the diesel engine–powered fire apparatus.^8,10^ DEE has been shown to infiltrate station environments when not properly contained, compounding long-term exposure risk.^8,10,11^ Three studies evaluated PFAS-containing firefighting foams.^12,13,41,48^ Three others explored contamination from turnout gear, a direct pathway to dermal and respiratory exposures.^19,20,44^ Two other studies have shown that the turnout gear itself, which consists of multiple layers and materials, contains PFAS.^25,26^

#### 3.3.3. Basic preventive measures

All studies addressing basic preventive measures identified proper PPE as the primary line of defense against occupational exposure.^1,12,46,49^ Proper PPE consists of full turnout gear and wearing a self-contained breathing apparatus as much as possible. Other powerful tools include training and awareness of occupational cancer risks. Four articles highlighted the importance of regular awareness training, routine medical evaluations, and cancer screenings for firefighters.^29,37,45,50^ When departments implement these practices consistently, they increase the likelihood of detecting cancer symptoms at earlier, more treatable stages.^51^ Two articles examined the possibility of substitution of PFAS-containing firefighting foams with fluorine-free firefighting foams.^40,42^

Although not all studies addressed global frameworks, the IARC report emphasizes the critical role of prevention in reducing cancer burden, particularly through the use of PPE and early decontamination practices.^14^ The IARC Working Group recommendations stress that exposure minimization strategies, when consistently applied, can significantly reduce occupational risk even when total elimination is not possible.^14^ This reinforces the literature’s emphasis on showering post-fire, PPE cleaning, and on-scene gross decontamination.

#### 3.3.4. Effectiveness of clean PPE

Four studies detailed the effectiveness of clean PPE.^19,20,27,29^ Although this topic could fall under the theme of basic preventive measures, it was identified as a separate focus in the analysis of the articles. This distinction stems from the entrenched fire service culture where dirty gear is often regarded as a badge of honor.^20^ This dangerous mindset is directly linked to the elevated cancer incidence and mortality rates among firefighters.^44^ Six studies suggested constantly washing all gear and equipment after incidents when exposed to potential carcinogens, such as those from fires.^5,19,20,29,52,53^ Four records suggested firefighters shower within an hour of being exposed to fire and change into clean station clothes.^29,37,52,53^

#### 3.3.5. Station designs and decontamination models

Some studies provided information for improved station designs and decontamination models. Station designs and decontamination models can also be considered preventive measures, but they require more planning and research and should not be considered a “basic” preventive measure. Three studies and training videos emphasized the importance of fire station design in reducing carcinogenic exposure by ensuring turnout gear and firefighting equipment are not stored in living quarters, and gear racks are positioned away from vehicle exhaust systems.^8,10,45^ These engineering controls are critical for minimizing ambient contamination within the station environment. However, despite these established guidelines, many fire departments continue to fall short in implementing these basic preventive measures, underscoring the need for ongoing education and enforcement.^54^ Additionally, two studies examined exhaust fans for fire trucks to reduce exposure to DEE.^8,10^ Two records described a decontamination model to reduce the tracking of carcinogens from the fire scene back to the fire station.^29,53^

Whereas most models are national or department-based, the broader guidance from IARC indirectly supports the need for environmental and engineering controls that minimize airborne and dermal exposures in station settings.^14^ The classification of firefighting as a Group 1 carcinogenic occupation reinforces the urgency of implementing clean cab designs, turnout gear storage protocols, and station zoning to reduce chronic low-level exposures.

## 4. Discussion

### 4.1. Occupational exposure and prevention: key insights from the literature

This systematic review confirms a strong occupational link between firefighting and elevated cancer incidence and mortality, supported by consistent international findings. For instance, according to the International Association of Fire Fighters (IAFF), 72% of US and almost 94% of Canadian IAFF member deaths in 2023 were due to cancer.^55^ Studies across multiple cohorts demonstrate significantly increased risks for cancers, including multiple myeloma, testicular cancer, non-Hodgkin lymphoma, bladder cancer, mesothelioma, prostate cancer, and melanoma.^2–4^ These findings are echoed in the 2022 IARC Monograph that officially classified firefighting as a Group 1 carcinogenic occupation due to sufficient evidence of carcinogenicity in humans. Table 1 summarizes the types of cancers identified in the articles reviewed, the body systems affected, and whether they were found to be significantly elevated. These cancer types align closely with the occupational exposures detailed earlier. Where available, quantitative effect estimates (Standardized Mortality Ratio (SMR), Standardized Incidence Ratio (SIR), and Odds Ratio (OR)) and corresponding 95% CIs from the original epidemiological studies are reported in Table S9. Cancer-causing exposures in firefighting are multifactorial and originate from diverse sources. Carcinogenic combustion products such as benzene, formaldehyde, hydrogen cyanide, dioxins,^56^ and PAHs result from the burning of synthetic building materials,^7^ insulation, flooring, and electrical cables.^7,57,58^ DEE, a Group 1 carcinogen, adds chronic exposure risk in fire stations that lack adequate exhaust capture systems.^8^ Additionally, PFAS found in AFFF firefighting foams and turnout gear pose long-term risks through dermal absorption and off-gassing.^59^

**Table 1:** Summary of cancer types and body systems affected among firefighters in reviewed literature.

Type of cancer	Affected body area	Statistically significant increase in risk	References
**Adenocarcinoma of the esophagus**	Food pipe (esophagus)	Yes	Tsai RJ, et al, 2015^2^; Pinkerton L, et al, 2020^4^
**Lung cancers**	Lungs	Yes	Tsai RJ, et al, 2015^2^; Pinkerton L, et al, 2020^4^
**Leukemia**	Blood	Yes	Tsai RJ, et al, 2015^2^; Pinkerton L, et al, 2020^4^
**Kidney cancer**	Kidney	Yes	Tsai RJ, et al, 2015^2^; Pinkerton L, et al, 2020^4^
**Multiple myeloma**	Bone marrow	Yes	Tsai RJ, et al, 2015^2^; Daniels RD, 2014^3^; Pinkerton L, et al, 2020^4^
**Tongue cancer**	Tongue	Yes	Tsai RJ, et al, 2015^2^
**Testicular cancer**	Testicles	Yes	Tsai RJ, et al, 2015^2^; Daniels RD, 2014^3^; Pinkerton L, et al, 2020^4^; Demers PA, et al, 2022^11^; Jalilian H, et al, 2019^5^
**Bladder cancer**	Bladder	Yes	Tsai RJ, et al, 2015^2^; Pinkerton L, et al, 2020^4^; Demers PA, et al, 2022^11^; Jalilian H, et al, 2019^5^
**Non-Hodgkin lymphoma (NHL)**	Lymphatic system	Yes	Tsai RJ, et al, 2015^2^; Daniels RD, 2014^3^; Pinkerton L, et al, 2020^4^; Demers PA, et al, 2022^11^; Jalilian H, et al, 2019^5^
**Gum, lip, and other mouth cancer**	Mouth	No	Tsai RJ, et al, 2015^2^
**Pharyngeal cancer**	Throat	No	Tsai RJ, et al, 2015^2^
**Esophageal squamous carcinoma**	Food pipe (esophagus)	No	Tsai RJ, et al, 2015^2^
**Stomach cancer**	Stomach	Yes	Tsai RJ, et al, 2015^2^; Daniels RD, 2014^3^; Pinkerton L, et al, 2020^4^
**Liver cancer**	Liver	No	Tsai RJ, et al, 2015^2^; Daniels RD, 2014^3^; Pinkerton L, et al, 2020^4^
**Pancreatic cancer**	Lower stomach (pancreas)	No	Tsai RJ, et al, 2015^2^
**Soft tissue sarcoma**	Soft tissue (muscle, tendons, fat, lymph, blood vessels, nerves)	No	Tsai RJ, et al, 2015^2^
**Mesothelioma**	Lungs	Yes	Tsai RJ, et al, 2015^2^; Pinkerton L, et al, 2020^4^; Demers PA, et al, 2022^11^; Jalilian H, et al, 2019^5^
**Thyroid cancer**	Thyroid (gland at the base of the neck)	Yes	Tsai RJ, et al, 2015^2^; Demers PA, et al, 2022^11^; Jalilian H, et al, 2019^5^
**Hodgkin lymphoma**	Lymphatic system	No	Tsai RJ, et al, 2015^2^
**Colon**	Colon or rectum	Yes	Tsai RJ, et al, 2015^2^; Daniels RD, 2014^3^; Pinkerton L, et al, 2020^4^; Demers PA, et al, 2022^11^; Jalilian H, et al, 2019^5^
**Rectal**	Rectum	Yes	Daniels RD, 2014^3^; Pinkerton L, et al, 2020^4^; Jalilian H, et al, 2019^5^
**Prostate**	Prostate	Yes	Daniels RD, 2014^3^; Pinkerton L, et al, 2020^4^; Demers PA, et al, 2022^11^; Jalilian H, et al, 2019^5^
**Malignant melanoma**	Skin	Yes	Daniels RD, 2014^3^; Demers PA, et al, 2022^11^; Jalilian H, et al, 2019^5^
**Brain**	Brain	Yes	Daniels RD, 2014^3^

‘Yes’ indicates that the study reported a statistically significant increase in cancer risk (e.g., relative risk, odds ratio, or standardized mortality/incidence ratio). ‘No’ indicates that the increase in risk was not statistically significant, although some studies may still report elevated (but non-significant) risk estimates.

Despite this well-established exposure profile, current prevention strategies remain inconsistently implemented. Cultural norms within fire departments often perpetuate dangerous behaviors. For example, the tradition of treating soiled gear as a “badge of honor” continues to hinder regular decontamination and PPE hygiene practices.^20^ Research by Wolffe et al^30^ revealed that peer norms, organizational hierarchy, and identity narratives play critical roles in firefighters’ risk perception and compliance with decontamination procedures. Addressing cultural inertia is therefore essential for any prevention strategy.

Training programs and institutional policies often prioritize career departments, leaving volunteer firefighters underprepared and underprotected.^60^ Volunteers, who make up over 65% of the US firefighting workforce, often lack access to gear extractors, second sets of PPEs, dedicated decontamination zones, or on-site cancer screening.^15^ Firefighters responding from home in personal vehicles face unique challenges, including transporting contaminated gear near groceries, family members, and personal items, which can extend the chain of exposure.^19,20^ Furthermore, few occupational cancer incidence studies include volunteer cohorts, creating data gaps that obscure the full scope of cancer burden in the fire service.^61,62^

Although several robust decontamination protocols, such as the Swedish Skellefteå model, exist, these are primarily designed for firehouses with extensive infrastructure and resources.^29^ The hierarchy of controls, from elimination to PPE, must be operationalized differently depending on the context. Eliminating PFAS-based foams, installing diesel exhaust capture systems, improving ventilation, and standardizing gear hygiene are all critical measures, but are adopted unevenly.

Policy-level changes are also necessary. Cancer surveillance should include mandatory reporting of firefighter diagnoses across all departments, including volunteers. Legislative support for fluorine-free firefighting foams and PPE reformulation is needed to address PFAS-related risks. Until these structural changes occur, firefighters, especially volunteers, remain vulnerable.

### 4.2. Schutte Decontamination Model: a proposed volunteer-specific decontamination framework

The Schutte Decontamination Model integrates components that are supported by existing empirical evidence with context-specific adaptations proposed by the investigators to address gaps faced by volunteer firefighters responding in personal vehicles. Evidence-based elements include post-fire wet decontamination, PPE hygiene practices, clean cab principles, and prompt skin cleaning, all of which are supported by prior studies^8,10,19,20,45^ and established models such as the Skellefteå model.^29^ In contrast, the application of sealed rolling storage units for transport in personal vehicles represents a proposed, theory-informed adaptation designed to extend these principles to decentralized firefighting contexts.

In response to the systemic gaps identified in the literature, we propose the Schutte Decontamination Model, a pragmatic and low-cost framework designed to meet the needs of volunteer firefighters who respond from home using personal vehicles. Unlike existing decontamination models developed for career departments with centralized infrastructure, the Schutte Model is tailored to resource-limited, decentralized fire services, as there are currently no published decontamination models addressing firefighters who directly respond to emergencies using their POVs.^29,52,53^

The model builds upon key principles from the award-winning Skellefteå model, particularly the “clean cab” concept, which seeks to isolate contaminated gear from clean areas.^29^ However, Schutte’s approach adapts these principles to the rural US context, where fire stations may lack ventilation systems, second sets of gear, or turnout gear washers. The key challenge addressed is the unsafe transport of contaminated gear in POVs, which creates prolonged exposure risk for both firefighters and their families. Key components of Schutte’s Model include the following:

#### 4.2.1. Sealed storage units

**Figure 2:**
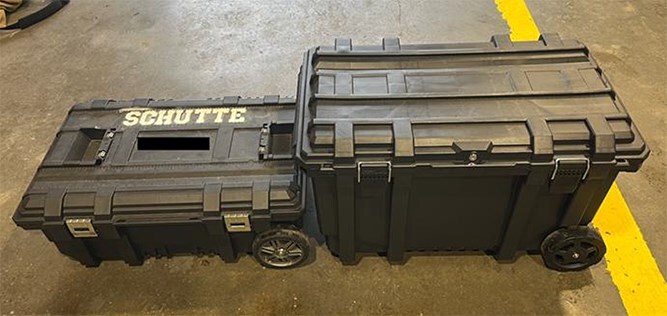
Rolling toolboxes for gear storage and transportation.

**Figure 3:**
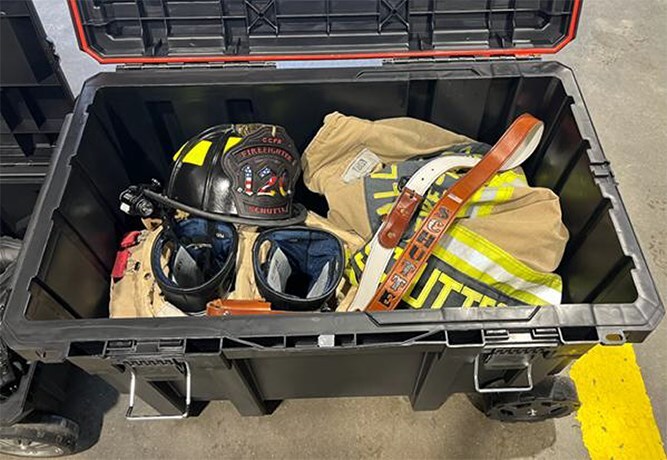
Larger box accommodates gear intact.

Recent studies have shown even “cleaned” turnout gear can retain carcinogenic PFAS under typical conditions, continuing to pose exposure risks.^25,26^ This underscores the importance of proper storage, both when transporting clean gear to a fire scene and when returning with contaminated gear. Volunteer firefighters, in particular, face the challenge of transporting their gear in personal vehicles, often using mesh or nylon bags that provide little protection against off-gassing. This not only undermines the clean cab principle but also poses serious health risks to the firefighters and their families. To address this issue, the Schutte Model incorporates the use of durable, hard-plastic rolling toolboxes (25- and 50-gallon (approximately 95L and 189L)) with water- and dust-sealed lids (Figure 2). These containers provide a near-airtight environment, limiting carcinogen spread while supporting both deployment readiness and safe return transport. Whereas the 50-gallon box accommodates fully assembled gear (Figure 3), the smaller 25-gallon box requires gear disassembly, which may affect emergency response time. Nevertheless, both options present cost-effective, practical solutions for minimizing secondary contamination.

#### 4.2.2. Post-fire decontamination protocol

The model emphasizes the importance of immediate, on-scene wet decontamination following a fire. Firefighters are instructed to rinse down while still in their turnout gear using a cleaning solution and a brush.^20^ This initial scrub reduces surface contaminants before gear removal. The firefighter must then remove their gear carefully to avoid skin contact with contaminated materials, ideally with assistance or while wearing medical gloves. To further reduce inhalation risk during gear handling, a respirator (eg, N95 or higher) should be worn. Additionally, both gloves and masks should be stored in the same sealed box for quick access post-incident.

#### 4.2.3. Secondary safety measures

After gear removal, firefighters should clean exposed skin areas such as the face, neck, and hands with wipes. All contaminated gear is then placed inside a plastic bag, sealed with a zip tie, and stored in the sealed toolbox. Because volunteer firefighters often do not return directly to a fire station, the model includes flexible options: either remove contaminated clothing and change into clean coveralls or wear the coveralls over the base layer until clothing can be safely laundered. Contaminated clothing, if not removed immediately, should be isolated and handled with the same precautions as turnout gear.

#### 4.2.4. Personal protection and clothing management

Coveralls are essential to this protocol and are stored in the sealed container as part of the standard kit. After completing gear handling and changing, the firefighter can safely transport the storage box in their POV. Ideally, contaminated gear should be returned using fire department apparatus, but in many rural or volunteer contexts, this is not practical. Once home or back at the station, turnout gear should be cleaned following the Skellefteå model, and personal clothing should be washed separately to avoid household contamination. The goal is to create a realistic yet protective system that reduces the transport of carcinogens in personal vehicles.

### 4.3. Enhancing safety with the Schutte Model

**Figure 4:**
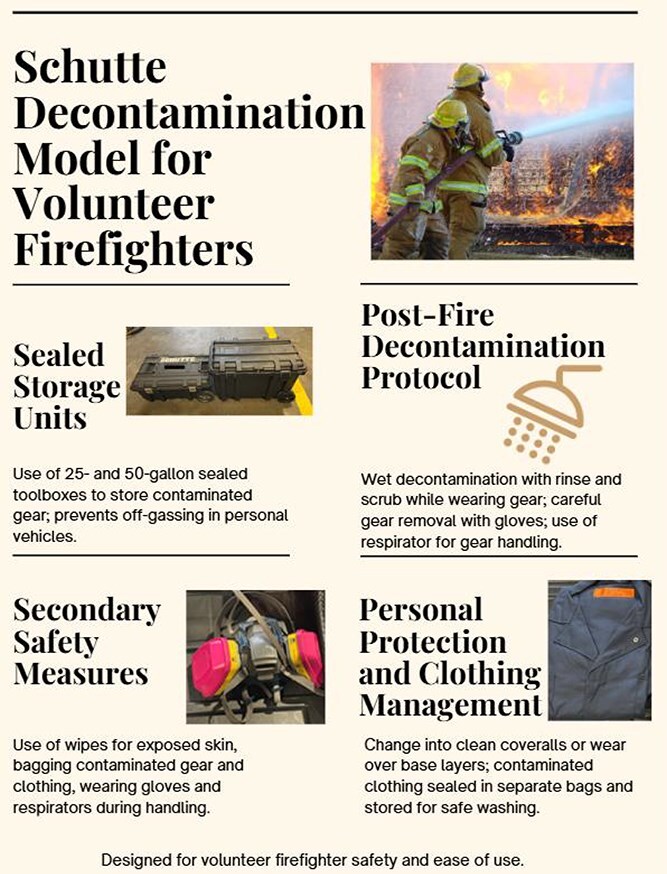
Key components of the proposed Schutte Decontamination Model for volunteer firefighters.

The Schutte Model offers a practical, low-cost, and evidence-informed approach to preventing secondary contamination, particularly tailored to the realities of volunteer firefighting. With an estimated implementation cost of just $154–$184, departments can equip personnel with simple yet effective tools to safely transport contaminated gear without compromising response time. The cost estimates are based on current US market prices and may vary by region and over time. See Table S10 and Figure S1 for a list of required items. Unlike gear bans in personal vehicles, which may delay rural emergency responses, the model upholds the clean cab principle while preserving operational flexibility. By combining sealed gear storage, post-fire hygiene protocols, and protective clothing practices into a unified system, the Schutte Model addresses a longstanding gap in occupational health protections for decentralized firefighting services. Figure 4 provides an infographic that explains the Schutte Model.

In conclusion, the Schutte Model strengthens firefighter cancer prevention efforts by targeting overlooked exposure risks in volunteer departments. Combined with the findings of this systematic review, it emphasizes the urgent need for inclusive, adaptable, and resource-sensitive decontamination strategies that extend occupational protections to all firefighters, regardless of setting, structure, or funding.

The proposed prevention strategies can be explicitly aligned with the Occupational Health and Safety Administration (OSHA) hierarchy of controls. Elimination and substitution are represented by the transition toward fluorine-free firefighting foams and PFAS-free PPE materials. Engineering controls include diesel exhaust capture systems, improved station ventilation, and sealed gear containment to isolate contaminated equipment from vehicle interiors. Administrative controls encompass decontamination protocols, training, and procedural changes, while PPE remains a critical final layer of protection. The Schutte Model primarily operationalizes administrative and PPE controls in contexts where higher-order controls are not yet feasible, particularly in volunteer and rural fire services.

Although the Schutte Model was developed with rural US volunteer firefighters in mind, its core principles may be applicable to other contexts, including urban volunteer departments and international fire services where firefighters respond using personal or mixed-use vehicles. Adaptation may be required in settings with different vehicle regulations, storage standards, or occupational safety policies. Nevertheless, the model’s emphasis on containment, separation, and early decontamination provides a flexible framework that can be modified to align with local operational and regulatory environments.

### 4.4. Study strengths and limitations

This systematic review has several notable strengths. It represents one of the few comprehensive syntheses addressing both occupational cancer risks and preventive strategies among firefighters, integrating evidence from peer-reviewed studies, international reports, and training materials. Furthermore, the development of the Schutte Model offers a novel, low-cost, and context-specific intervention tailored to the unique needs of volunteer firefighters who respond using personal vehicles. This practical model bridges an important gap in existing decontamination and cancer prevention frameworks.

Despite these strengths, several limitations should be acknowledged. First, although a systematic search strategy was employed, publication bias cannot be ruled out, as unpublished reports may have been missed. Second, the review was limited to English language publications, which may have excluded relevant studies published in other languages and introduced potential language bias, particularly given the international nature of the firefighting workforce. The included literature also varied widely in study design, population characteristics, and exposure assessment methods, limiting direct comparisons and preventing quantitative meta-analysis. Additionally, heterogeneity between career and volunteer firefighter populations, along with differences in international firefighting practices, complicate the generalizability of the findings. Finally, although the proposed Schutte Model was developed based on existing best practices, it has not yet been empirically validated in real-world field conditions.

### 4.5. Future research directions

Although this review highlights significant progress in understanding cancer risks among firefighters, several areas require further exploration to strengthen prevention efforts and inform policy. First, future studies should include volunteer firefighters as a distinct study population. Despite comprising the majority of the fire service in countries like the United States and parts of Europe, this group remains significantly underrepresented in cancer incidence and exposure studies. Large-scale epidemiological research distinguishing between career and volunteer firefighters could help clarify differential risks and inform tailored interventions.

Second, research should focus on validating the Schutte Model through pilot studies that assess its real-world effectiveness in reducing carcinogen transfer to personal vehicles, clothing, and homes, particularly in volunteer departments lacking centralized facilities. These studies should evaluate the feasibility of adoption, contamination levels before and after implementation, and firefighters’ perceptions of the model’s practicality. Additionally, cost-effectiveness analyses of interventions such as sealed gear storage, enhanced training, and upgraded PPE are needed to compare implementation costs with potential long-term health care savings, providing evidence to support institutional and policy-level adoption.

Moreover, more integrative research is needed that links chemical exposure data with biological markers, long-term health outcomes, and psychosocial factors (eg, awareness, behavior change, organizational support). Expanding the use of wearable sensors, real-time exposure monitors, and health surveillance tools may offer innovative avenues to close existing gaps.

Finally, there is a need to establish and disseminate standardized decontamination protocols that are feasible across departments of varying sizes and budgets. These protocols should be supported by formal training modules and reinforced through ongoing firefighter education and leadership engagement.

## 5. Conclusions

This study aimed to systematically examine key cancer-related risks in firefighting by synthesizing evidence across 3 critical domains: occupational exposures, cancer incidence and mortality, and available prevention strategies. While the scope of exposures in the fire service is vast and constantly evolving, this review offers a consolidated overview of major hazards, including carcinogenic combustion byproducts, DEE, PFAS-containing gear and foams, and inadequate decontamination practices. Notably, we also propose a novel, practical decontamination model explicitly designed for volunteer firefighters in rural settings, an often-overlooked population in cancer prevention efforts.

Although it is not feasible to capture every exposure pathway in a single study, this work contributes to the field by integrating multiple strands of evidence, epidemiological trends, preventive practices, and context-specific solutions into one comprehensive framework. The cancer types most frequently associated with firefighting include multiple myeloma, testicular cancer, non-Hodgkin lymphoma, bladder, colon, and prostate cancer, mesothelioma, and melanoma, reinforcing the need for targeted prevention measures. Strategies such as improving PPE use, implementing clean cab protocols, redesigning fire stations, and increasing awareness through training are identified as critical components of cancer risk reduction.

Ultimately, firefighter cancer prevention depends not only on technological or procedural change but also on a cultural shift toward proactive health and safety practices. Using the OSHA hierarchy of controls as a guide, the findings underscore the potential to eliminate, substitute, engineer out, or, at a minimum, administratively control many of these hazards. This review, although not exhaustive, fills a critical gap by addressing exposures, outcomes, and prevention in an integrated manner and offers a replicable decontamination model tailored to under-resourced fire departments.

## Supplementary Material

Supplemental_Materials_Tables_and_Figures_Final_01182026_uiag013

## Data Availability

The data that support the findings of this study are available in Table S1. All the articles can be found in the databases used for this study.
