# Supplementary material for: Firefighter cancer risks: a systematic review and proposal for a volunteer-specific decontamination model
Source: J Occup Health. 2026 Mar 7;68(1):uiag013. doi: 10.1093/joccuh/uiag013 (PMC13125747; doi:10.1093/joccuh/uiag013)
Supplement: Supplemental_Materials_Tables_and_Figures_Final_01182026_uiag013 [file supplemental_materials_tables_and_figures_final_01182026_uiag013.docx]

**Supplementary Tables and Figures**

**Supplementary Table 1:** Description of included studies, topics discussed, and quality assessment score

| S/N | Author(s), Year | Country | Type | Paper Title | Topics Discussed | Quality Assessment Score |
| --- | --- | --- | --- | --- | --- | --- |
| 1 | Tsai, R. J. et al., 2015 | USA | A | Risk of cancer among firefighters in California, 1988–2007 | I, II | 8/10 |
| 2 | Daniels RD, 2014 | USA | A | Mortality and cancer incidence in a pooled cohort of US firefighters from San Francisco, Chicago and Philadelphia (1950–2009) | I | 8/11 |
| 3 | Pinkerton L, et al., 2020 | USA | A | Mortality in a cohort of US firefighters from San Francisco, Chicago and Philadelphia: an update. | I | 8/11 |
| 4 | Demers PA, et al., 2022 | USA | A | Carcinogenicity of occupational exposure as a firefighter. | I, II, IV | 7/10 |
| 5 | Chung J et al., 2020 | Canada | A | Career fire hall exposures to diesel engine exhaust in Ontario, Canada | II, III | 5/8 |
| 6 | Rosenfeld Pem et al. 2023 | USA | A | Perfluoroalkyl substances exposure in firefighters: Sources and implications. | II | 9/12 |
| 7 | Mazumder NU, et al. 2023 | USA | A | Firefighters’ exposure to per-and polyfluoroalkyl substances (PFAS) as an occupational hazard: a review | II | 9/12 |
| 8 | Jalilian H, et al., 2019 | Iran | A | Cancer incidence and mortality among firefighters | I, II | 6/10 |
| 9 | NFPA, 2022 | USA | A | U.S. Fire Department Profile | I | 6/6 |
| 10 | Tonnaer C, 2019 | Netherlands | A | Legal status of (on-call) volunteer firefighters in Europe | V | 5/6 |
| 11 | Morris CE, et al., 2022 | USA | A | Comparing physical fitness in career vs. volunteer firefighters. | III | 7/8 |
| 12 | Aliano-Gonzalez MJ, et al., 2022 | Spain | A | Assessment of volatile compound transference through firefighter turnout gear. | II, IV | 6/8 |
| 13 | Harrison, T., et al., 2017 | USA | A | Resilience, culture change, and cancer risk reduction in a fire rescue organization: Clean gear as the new badge of honor | I, II, III, IV, V | 8/10 |
| 14 | Maizel A, et al., 2023 | USA | A | Per-and polyfluoroalkyl substances in new firefighter turnout gear textiles. | II | 6/8 |
| 15 | Maizel A, et al., 2024 | USA | A | Per-and Polyfluoroalkyl Substances in Firefighter Turnout Gear Textiles Exposed to Abrasion, Elevated Temperature, Laundering, or Weathering | II | 7/8 |
| 16 | Peaslee, G., et al., 2020 | USA | A | Another pathway for firefighter exposure to per and polyfluoroalkyl substances: firefighetr textiles | II | 7/8 |
| 17 | Magnusson S., et al., 2015 | Sweden | A | Healthy firefighters - the Skelleftea Model improves the work environment | I, II, III, IV, V | 5/6 |
| 18 | Baldwin, et al., 2011 | USA | A | Controlling diesel exhaust exposure inside firehouses | II, III, V | 6/6 |
| 19 | Fire and Emergency NZ, 2014 | New Zealand | V | Preventing Firefighter Cancer | I, II, III, IV, V | 4/6 |
| 20 | Ganesan K, et al., 2010 | India | A | Chemical warfare agents | II | 7/12 |
| 21 | Hsu JF, et al., 2011 | Taiwan | A | An occupational exposure assessment of polychlorinated dibenzo-p-dioxin and dibenzofurans in firefighters. | II | 6/8 |
| 22 | Stellman SD, et al., 2004 | USA | A | Exposure opportunity models for Agent Orange, dioxin, and other military herbicides used in Vietnam, 1961–1971 | II | 6/8 |
| 23 | Kaller M, et al., 2023 | USA | A | An evaluation of the firefighting performance of alcohol-resistant aqueous film forming foams (AFFF-AR) and alcohol-resistant fluorine-free foams (FFF-AR) in the past two decades | II, III | 5/8 |
| 24 | Chemguard, 2005 | USA | A | General foam information | II | 5/6 |
| 25 | IAFF, 2024 | USA | A | Fire fighter cancer awareness month | I | 5/6 |
| 26 | MU Fire and Rescue Training Institute, 2016 | USA | V | The Silent Killer - Firefighter Cancer | I, II, III, IV, V | 5/6 |
| 27 | US Department of Labor, 2023 | USA | A | Information related to Federal Firefighter claims | I | 4/6 |
| 28 | First Responder Center of Excellence, 2018 | USA | V | Firefighter Cancer Inititative | I, II, III, IV, V | 6/6 |
| 29 | Betus, M., et al., 2023 | Slovakia | A | Ensuring firefighter safety and resource preservaton from contamination in demolition building fires | II, IV | 5/5 |
| 30 | City of Concord, 2016 | USA | V | The Concord Decontamination Model | V | 6/6 |
| 31 | Back , G., et al., 2020 | USA | A | An evaluation of the firefighting effectiveness of fluorine free foams | II, III | 5/8 |
| 32 | Dubocq, F., et al., 2020 | Sweden | A | Characterization of the chemcial contents of fluorintaed and flourine free firefighting foams using a novel worklfow combining nontarget screening and total flourine analysis | II, III | 5/8 |
| 33 | Laroche, E., et al., 2021 | Canada | A | Cancer incidence and mortality among firefighters: an overview of epidemiologic systematic reviews | I, II | 6/10 |
| 34 | Forester, C., et al., 2022 | USA | A | Effects of temperature and advanced cleaning practices on the removal of select organic chemicals from structural firefighting gear | III, IV | 6/8 |
| 35 | Department of Health and Human Services, 2004 | USA | A | A summary of health hazard evaluations: Issues related to occupational exposure to fire fighters, 1990 to 2001 | II | 6/6 |
| 36 | Stec, A., et al., 2018 | UK | A | Occupational exposure to polycyclic aromatic hydrocarbons and elevated cancer incidence in firefighters | II | 5/8 |
| 37 | IARC, 2023 | International organization | A | Occupational Exposure as a Firefighter | I, II, III, V | 7/10 |

Note topics to be (I) Cancer incidence and mortality; (II) Cancer exposure; (III) Basic preventive measures; (IV) Effectiveness of clean personal protective equipment; and (V) Station designs and decontamination models. The record types are (A) article and (V) Video.

**Supplementary Table 2: Quantitative Effect Estimates for Firefighter Cancer Outcomes Reported in Included Studies**

| **Study** | **Cancer Type** | **Effect Measure** | **Estimate** | | **95% CI** | | **Comparison Group** |
| --- | --- | --- | --- | --- | --- | --- | --- |
|  |  |  | **Cancer Incidence** | **Mortality** | **Cancer Incidence** | **Mortality** |  |
| Daniels et al., 2014 | All cancer (mortality ) | SIR/ SMR | 1.09 | 0.99 | 1.06 to 1.12 | 0.97-1.01 | US General Population |
|  | Esophageal cancer | SIR/SMR | 1.6 | 1.39 | 1.31-2.00 | 1.14-1.67 | US General Population |
|  | Multiple myeloma | SIR/SMR | 72 | 0.89 | 0.50- 0.99 | 0.64-1.20 | US General Population |
|  | Stomach Cancer |  | 1.15 | 1.10 | 0.93-1.40 | 0.91-1.33 | US General Population |
|  | Testicular cancer | SIR /SMR | 0.75 | 0.73 | 0.42-1.24 | 0.15- 2.14 | US General Population |
|  | Malignant mesothelioma | SMR | 2.29 | 2.00 | 1.60-3.19 | 1.03- 3.49 | US General Population |
|  |  |  |  |  |  |  |  |
| Tsai et al., 2015 | **Head and neck** |  |  |  |  |  |  |
|  | Lip | OR | 1.44 |  | 0.89-2.33 |  | US California population |
|  | Tongue | OR | 1.18 |  | 0.82-1.70 |  | US California population |
|  | Salivary gland | OR | 1.30 |  | 0.75-2.25 |  | US California population |
|  | Gum and other mouth | OR | 1.07 |  | 0.62-1.85 |  | US California population |
|  | Pharyngeal | OR | 1.06 |  | 0.75-1.50 |  | US California population |
|  | **Digestive** |  |  |  |  |  | US California population |
|  | Esophagus | OR | 1.59 |  | 1.20-2.09 |  | US California population |
|  | Esophagus-adenocarcinoma | OR | 1.85 |  | 1.34-2.55 |  | US California population |
|  | Esophagus-squamous carcinoma | OR | 0.96 |  | 0.53-1.73 |  | US California population |
|  | Stomach | OR | 0.81 |  | 0.59-1.11 |  | US California population |
|  | Colorectal | OR | 1.10 |  | 0.93-1.31 |  | US California population |
|  | Liver | OR | 1.07 |  | 0.75-1.53 |  | US California population |
|  | Pancreas | OR | 1.10 |  | 0.83-1.46 |  | US California population |
|  | **Respiratory** |  |  |  |  |  | US California population |
|  | Larynx | OR | 0.59 |  | 0.39-0.89 |  | US California population |
|  | Lung and bronchus | OR | 1.08 |  | 0.92-1.28 |  | US California population |
|  | Lung-adenocarcinoma | OR | 1.10 |  | 0.89-1.35 |  | US California population |
|  | Lung-squamous cell | OR | 0.89 |  | 0.69-1.14 |  | US California population |
|  | Lung-small cell | OR | 1.24 |  | 0.95-1.61 |  | US California population |
|  | Lung-large cell | OR | 0.84 |  | 0.55-1.28 |  | US California population |
|  | Lung-non-specific non-small cell cancer | OR | 2.01 |  | 1.38-2.93 |  | US California population |
|  | **Connective tissue/skin** |  |  |  |  |  | US California population |
|  | Soft tissue, including the heart | OR | 1.16 |  | 0.76-1.77 |  | US California population |
|  | Melanoma | OR | 1.75 |  | 1.44-2.13 |  | US California population |
|  | Mesothelioma | OR | 1.40 |  | 0.89-2.21 |  | US California population |
|  | **Urinary/reproductive** |  |  |  |  |  | US California population |
|  | Prostate | OR | 1.45 |  | 1.25-1.69 |  | US California population |
|  | Testis | OR | 1.10 |  | 0.73-1.66 |  | US California population |
|  | Urinary bladder | OR | 0.99 |  | 0.78-1.26 |  | US California population |
|  | Kidney | OR | 1.27 |  | 1.01-1.59 |  | US California population |
|  | **Cranial/endocrine** |  |  |  |  |  | US California population |
|  | Brain | OR | 1.54 |  | 1.19-2.00 |  | US California population |
|  | Thyroid | OR | 1.27 |  | 0.88-1.84 |  | US California population |
|  | Hodgkin lymphoma | OR | 1.15 |  | 0.72-1.83 |  | US California population |
|  | Non-Hodgkin lymphoma | OR | 1.22 |  | 1.00-1.50 |  | US California population |
|  | Multiple myeloma | OR | 1.35 |  | 1.00-1.82 |  | US California population |
|  | Leukemia | OR | 1.32 |  | 1.05^1.66 |  | US California population |
|  |  |  |  |  |  |  |  |
| Demers et al., 2022 | NOT CALCULATED AND REPORTED IN THE STUDY | | | | | | |
| Pinkerton et al., 2020 | All Cancers | SMR |  | 1.12 |  | 1.08 to 1.16 | US referent population |
|  | Mesothelioma | SMR |  | 1.86 |  | 1.10 -2.94 | US referent population |
|  | Non-Hodgkin Lymphoma | SMR |  | 1.21 |  | 1.03 - 1.42 | US referent population |
|  | cancers of the oesophagus | SMR |  | 1.31 |  | 1.10 – 1.55 | US referent population |
|  | intestine | SMR |  | 1.27 |  | 1.14 -1.40 | US referent population |
|  | rectum | SMR |  | 1.32 |  | 1.07 – 1.61 | US referent population |
|  | lung | SMR |  | 1.08 |  | 1.02 – 1.15 | US referent population |
|  | kidney | SMR |  | 1.22 |  | 1.00 – 1.47 | US referent population |
|  | Cancers of the buccal cavity and pharynx | SMR |  | 1.35 |  | 1.11 – 1.63 | US referent population |
|  | Biliary, liver, and gallbladder | SMR |  | 1.36 |  | 1.15 – 1.60 | US referent population |
|  | Unspecified digestive cancers | SMR |  | 1.64 |  | 1.02 -2.51 | US referent population |
|  | Cancer of the bladder | SMR |  | 0.98 |  | 0.80 – 1.18 | US referent population |
|  | Prostate | SMR |  | 1.08 |  | 0.97 - 1.20 | US referent population |
|  | Malignancy of the brain | SMR |  | 0.99 |  | 0.79 -1.23 | US referent population |
|  | Leukemia | SMR |  | 1.11 |  | 0.94 – 1.31 | US referent population |
|  | Multiple Myeloma | SMR |  | 0.93 |  | 0.70 – 1.21 | US referent population |
|  |  |  |  |  |  |  |  |
| Jalilian et al., 2019 | All cancers | SIRE /SMRE | 0.99 | 0.99 | 0.93 – 1.05 | 0.92 – 1.06 | US referent population |
|  | Buccal cavity and pharynx | SIRE /SMRE | 1.15 | 1.21 | 0.91 – 1.44 | 0.95 -1.55 | US referent population |
|  | Esophagus | SIRE /SMRE | 1.09 | 1.01 | 0.87 – 1.37 | 0.76 – 1.34 | US referent population |
|  | Stomach | SIRE /SMRE | 1.04 | 1.03 | 0.90 – 1.20 | 0.92- 1.15 | US referent population |
|  | Intestine | SIRE | 1.27 | NA | 0.89 – 1.82 | NA | US referent population |
|  | Colon | SIRE /SMRE | 1.14 | 1.10 | 1.06 -1.23 | 0.91 – 1.34 | US referent population |
|  | Rectum | SIRE /SMRE | 1.09 | 1.36 | 1.00 -1.20 | 1.18 -1.57 | US referent population |
|  | Colo-rectal | SIRE /SMRE | 1.12 | 1.07 | 0.99 – 1.27 | 0.54 – 2.12 | US referent population |
|  | Liver/ gallbladder | SIRE /SMRE | 0.93 | 1.05 | 0.80 – 1.08 | 0.79 – 1.39 | US referent population |
|  | Pancreas | SIRE /SMRE | 1.09 | 1.13 | 0.96 – 1.24 | 0.99 – 1.29 | US referent population |
|  | Larnyx | SIRE /SMRE | 0.93 | 0.74 | 0.66 – 1.30 | 0.48 – 1.15 | US referent population |
|  | Lung | SIRE /SMRE | 0.94 | 1.00 | 0.84 – 1.06 | 0.92 – 1.09 | US referent population |
|  | Mesothelioma | SIRE | 1.60 | NA | 1.09 – 2.34 | NA | US referent population |
|  | Bone | SIRE | 1.05 | NA | 0.52 – 2.13 | NA | US referent population |
|  | Soft tissue sarcoma | SIRE | 1.11 | NA | 0.85 – 1.44 | NA | US referent population |
|  | Malignant melanoma | SIRE /SMRE | 1.21 | 1.33 | 1.02 -1.45 | 0.98 -1.81 | US referent population |
|  | Skin | SIRE /SMRE | 1.12 | 1.08 | 0.95 -1.31 | 0.79 -1.47 | US referent population |
|  | Breast | SIRE /SMRE | 1.02 | 2.47 | 0.47 – 2.25 | 0.65 -9.48 | US referent population |
|  | Prostate | SIRE /SMRE | 1.15 | 1.08 | 1.05 – 1.27 | 0.92 -1.27 | US referent population |
|  | Testis | SIRE | 1.34 | NA | 1.08 – 1.68 | NA | US referent population |
|  | Bladder | SIRE /SMRE | 1.12 | 1.22 | 1.04 – 1.21 | 0.93 -1.60 | US referent population |
|  | Kidney | SIRE /SMRE | 1.12 | 1.19 | 0.93 -1.36 | 0.90 -1.58 | US referent population |
|  | Eye | SIRE | 1.12 | NA | 0.59 – 2.48 | NA | US referent population |
|  | Brain and nervous system | SIRE /SMRE | 1.07 | 1.25 | 0.87 – 1.33 | 0.96 – 1.63 | US referent population |
|  | Thyroid | SIRE | 1.22 | NA | 1.01 – 1.48 | NA | US referent population |
|  | Non- Hodgkin’s lymphoma | SIRE /SMRE | 1.07 | 1.42 | 0.96 – 1.20 | 1.05 -1.90 | US referent population |
|  | Hodgkin’s disease | SIRE /SMRE | 1.12 | 1.21 | 0.86 – 1.47 | 0.46 – 3.18 | US referent population |
|  | Multiple myeloma | SIRE /SMRE | 1.00 | 1.07 | 0.83 – 1.23 | 0.83 – 1.37 | US referent population |
|  | Leukemia | SIRE /SMRE | 0.97 | 1.06 | 0.85 – 1.11 | 0.93 – 1.22 | US referent population |

SIR- Standardized Incidence Ratio; SMR – Standardized Mortality Ratio; OR – Odds Ratio; SIRE- Summary Incidence Risk Estimates; SMRE – Summary Mortality Risk Estimates; *Tsai et al.'s effect estimates were the odds ratios for various cancers among firefighters for all races combined.

**Supplementary Table 3:** List of Required Items

| **Required Items:** | **Cost (approx.)** |
| --- | --- |
| Safe Storage System 25 gal | $70 |
| Safe Storage System 50 gal | $100 |
| Coveralls | $37 |
| Roll of Heavy-Duty Plastic Trash Bags | $20 |
| Zip Ties (Bag of 100) | $10 |
| Medical Gloves | provided by department |
| Respirator / Filter Mask (Box of 10) | $17 |
| **Total** | **$154 - $184** |

**Supplementary Table 4:** Scores of case-control studies

| **STUDIES** | Tsai et al., 2015 |
| --- | --- |
| **JBI SCORE** | 8/10 |
| Were the groups comparable other than the presence of disease in cases or the absence of disease in controls? | YES |
| Were cases and controls matched appropriately? | YES |
| Were the same criteria used for identification of cases and controls? | YES |
| Was exposure measured in a standard, valid and reliable way? | YES |
| Was exposure measured in the same way for cases and controls? | YES |
| Were confounding factors identified? | NO |
| Were strategies to deal with confounding factors stated? | NO |
| Were outcomes assessed in a standard, valid and reliable way for cases and controls? | YES |
| Was the exposure period of interest long enough to be meaningful? | YES |
| Was appropriate statistical analysis used? | YES |

**Supplementary Table 5:** Scores of cohort studies

| **STUDIES** | Daniels, 2014 | Pinkerkton et al, 2020 |
| --- | --- | --- |
| **JBI SCORE** | 8/11 | 8/11 |
| Were the two groups similar and recruited from the same population? | YES | YES |
| Were the exposures measured similarly to assign people to both exposed and unexposed groups? | YES | YES |
| Was the exposure measured in a valid and reliable way? | YES | YES |
| Were confounding factors identified? | UNCLEAR | UNCLEAR |
| Were strategies to deal with confounding factors stated? | UNCLEAR | UNCLEAR |
| Were the groups/ participants free of the outcome at the start of the study (or at the moment of exposure)? | YES | YES |
| Were the outcomes measured in a valid and reliable way? | YES | YES |
| Was the follow up time reported and sufficient to be long enough for outcomes to occur? | YES | YES |
| Was the follow-up complete, and if not, were the reasons to loss to follow-up described and explored? | YES | YES |
| Were strategies to address incomplete follow up utilized? | NA | NA |
| Was appropriate statistical analysis used? | YES | YES |

**Supplementary Table 6:** Scores of cross-sectional studies

| **STUDIES** | Chung et al., 2020 | Morris et al., 2022 | Hsu et al., 2011 | Stellam et al., 2004 |
| --- | --- | --- | --- | --- |
| **JBI SCORE** | 5/8 | 7/8 | 6/8 | 6/8 |
| Were the criteria for inclusion in the sample clearly defined? | NO | UNCLEAR | YES | YES |
| Were the study subjects and the setting described in detail? | YES | YES | YES | YES |
| Was the exposure measured in a valid and reliable way? | YES | YES | YES | YES |
| Were objective, standard criteria used for measurement of the condition? | YES | YES | YES | YES |
| Were confounding factors identified? | NO | YES | N/A | N/A |
| Were strategies to deal with confounding factors stated? | NO | YES | N/A | N/A |
| Were outcomes measured in a valid and reliable way? | YES | YES | YES | YES |
| Was appropriate statistical analysis used? | YES | YES | YES | YES |

**Supplementary Table 7:** Scores of experimental/intervention studies

| **STUDIES** | Maizel et al., 2023 | Maizel et al, 2024 | Peaslee et al., 2020 | Kaller et al., 2023 | Betus et al., 2023 | Back et al., 2020 | Dubocq et al., 2020 | Forester et al., 2022 | Stec et al, 2018 | Aliano-Gonzalez et al., 2022 |  |
| --- | --- | --- | --- | --- | --- | --- | --- | --- | --- | --- | --- |
| **JBI SCORE** | 6/8 | 7/8 | 7/8 | 5/8 | 5/8 | 5/8 | 5/8 | 6/8 | 5/8 | 6/8 |  |
| Is it clear in the study what is the “cause” and what is the “effect”(i.e. There is no confusion about which variable comes first)? | YES | YES | YES | YES | YES | YES | YES | YES | YES | YES |  |
| Was there a control group? | YES | YES | YES | YES | NO | YES | NO | YES | UNCLEAR | YES |  |
| Were participants included in any comparisons similar? | YES | YES | YES | YES | YES | YES | YES | YES | YES | YES |  |
| Were there multiple measurements of the outcome, both pre and post the intervention/exposure?  Outcome 1-7 | NO | YES | YES | NO | YES | YES | NO | YES | YES | YES |  |
| Were the outcomes of participants included in any comparisons measured in the same way?  Outcome 1-7 | YES | YES | YES | YES | YES | UNCLEAR | YES | YES | YES | NO |  |
| Were outcomes measured in a reliable way?  Outcome 1-7 | YES | YES | YES | YES | YES | YES | YES | YES | YES | YES |  |
| Was follow-up complete and if not, were differences between groups in terms of their follow-up adequately described and analyzed? | N/A | N/A | UNCLEAR | UNCLEAR | N/A | N/A | NA | N/A | N/A | UNCLEAR |  |
| Was appropriate statistical analysis used? | YES | YES | YES | N/A | NO | N/A | YES | N/A | UNCLEAR | YES |  |

**Supplementary Table 8:** Scores of grey literatures

| **STUDIES** | NFPA,2022 | Tonnaer, 2019 | Fire and Emergency NZ, 2014(VIDEO) | CHEMGUARD, 2005 | IAFF,2024 | US Dept. of Labor, 2023 | First Responder Center, 2018 | Baldwin et al.,2011 | City of Concord, 2016 | Dept. of HHS, 2004 | Magnusson et al., 2015 | MU Fire Institute, 2016 |
| --- | --- | --- | --- | --- | --- | --- | --- | --- | --- | --- | --- | --- |
| **ACCODS SCORE** | 6/6 | 5/6 | 4/6 | 5/6 | 5/6 | 4/6 | 6/6 | 6/6 | 6/6 | 6/6 | 5/6 | 5/6 |
| (AUTHORITY)  Who is responsible for the intellectual content   Is it an individual author associated with a reputable organisation?  An individual author with professional qualifications or considerable experience?  An individual author who produced/published other work (grey/black) in the field ?  An individual author who is a recognized expert, identified in other sources?  Is it an individual author cited by others?(use google scholar as a quick check)  Is it an individual author who has higher degree student under “expert” supervision  Organization or group  Is the organisation reputable?  Is the organization an authority in the field?  In all cases  Does the item have a detailed reference list or bibliography? | YES | NO  YES  YES  YES  YES  NO    NO  YES  NO | UNLCEAR  NO  UNCLEAR  UNCLEAR  NO  NO  NO    PARTIAL YES  NO | NO    YES  YES  NO | UNCLEAR    YES  YES  NO | NO    YES  YES  NO | YES  YES  YES  YES  YES  YES  YES    NO | Yes | NO    YES   NO | YES  YES  YES  YES  YES    YES  YES  YES | YES | NA    YES  NO |
| (ACCURACY)   Does the item have a clearly stated aim or brief? Is so, is this met?    Does it have a stated methodology?  If so, is it adhered to?   Has it been peer-reviewed?  Has it been edited by a reputable authority?  Supported by authoritative, documented references or credible sources?   Is it representative of work in the field?   If No, is it a valid counterbalance?   Is any data collection explicit and appropriate for the research?   If item is secondary material (e.g. a policy brief of a technical report) refer to the original.   Is it an accurate, unbiased interpretation or analysis? | YES | YES  YES  YES  YES  YES  YES  YES    YES    YES  NA  YES | YES  NO  NO  NO  UNCLEAR  YES  NO  NA  PARTIAL YES | YES  NO  NO  NO  YES    YES | YES  NO  NO  NO  NO  YES  NO    PARTIAL YES | NA  NA  NA  NA  YES  NA | YES  NO  NO  NO  YES  YES  YES | Yes | YES  NO  NO  YES  NA  NA  NA | YES  YES  YES  YES  YES  YES  YES | UNCLEAR | NO  NO  NO  NO  NO  NO  YES  YES  NO  NA |
| (COVERAGE)   All items have parameters which define their content coverage. These limits might mean that a work refers to a particular population group, or that it excluded certain types of publication. A report could be designed to answer a particular question, or be based on statistics from a particular survey.  • Are any limits clearly stated? | YES | YES | YES | YES | YES | NO | YES | Yes | YES | YES | YES | YES |
| (OBJECTIVITY)   It is important to identify bias, particularly if it is unstated or unacknowledged.  • Opinion, expert or otherwise, is still opinion: is the author’s standpoint clear?   • Does the work seem to be balanced in presentation? | YES  NO | NO | YES  NO | YES  YES | YES  NO | YES  NO | YES  YES | Yes | YES  YES | YES  YES | YES | YES  YES |
| (DATE)  For the item to inform your research, it needs to have a date that confirms relevance  • Does the item have a clearly stated date related to content? No easily discernible date is a strong concern.  • If no date is given, but can be closely ascertained, is there a valid reason for its absence?  • Check the bibliography: have key contemporary material been included? | YES | YES      NO | NO  NO | YES    NO | YES    NO | YES    NA | YES    NA | Yes | YES  YES    NA | YES  YES    YES | YES | YES    NO |
| (SIGNIFICANCE)  This is a value judgment of the item, in the context of the relevant research area    • Is the item meaningful? (this incorporates feasibility, utility and relevance)  • Does it add context?   • Does it enrich or add something unique to the research?  • Does it strengthen or refute a current position?   • Would the research area be lesser without it?   • Is it integral, representative, typical?   • Does it have impact? (in the sense of influencing the work or behaviour of others) | YES | YES  YES  YES  YES  NO  YES  YES | YES  YES  YES  YES  YES  YES  YES | YES  YES  YES  YES  YES  YES  YES | YES  YES  YES  NO  YES | YES  NO  UNCLEAR  NA | YES  YES  YES  YES  YES | Yes | YES  YES  YES  YES  YES | YES  YES  YES  YES  YES | YES | YES  YES  YES  YES  YES  YES  YES |

**Supplementary Table 9:** Scores of narrative reviews

| **Studies** | Mazunder et al, 2023 | Ganesan et al,2010 | Rosenfield et al, 2022 |
| --- | --- | --- | --- |
| **SANRA SCORE** | 9/12 | 7/12 | 9/12 |
| Justification of the article’s importance for the readership | 2 | 1 | 2 |
| Statement of concrete aims or formulation of questions | 1 | 1 | 1 |
| Description of the literature search | 0 | 0 | 1 |
| Referencing | 2 | 2 | 2 |
| Scientific reasoning | 2 | 2 | 1 |
| Appropriate presentation of data | 2 | 1 | 2 |

**Supplementary Table 10:** Scores of systematic reviews/monographs

| STUDIES | Demers et al., 2022 | Laroche et al., 2021 | IARC., 2023 | Jalilian et al,   2019 | Harrison et al.,2017 |
| --- | --- | --- | --- | --- | --- |
| JBI SCORES | 7/10 | 6/10 | 7/10 | 6/10 | 8/10 |
| Is there congruity between the stated philosophical perspective and the research methodology? | YES | YES | YES | YES | YES |
| Is there congruity between the research methodology and the research question or objectives? | YES | YES | YES | YES | YES |
| Is there congruity between the research methodology and the methods used to collect data? | YES | YES | YES | YES | YES |
| Is there congruity between the research methodology and the representation and analysis of data? | YES | YES | YES | YES | YES |
| Is there congruity between the research methodology and the interpretation of results? | YES | YES | YES | YES | YES |
| Is there a statement locating the researcher culturally or theoretically? | UNCLEAR | N/A | UNCLEAR | N/A | UNCLEAR |
| Is the influence of the researcher on the research, and vice- versa, addressed? | UNCLLEAR | N/A | UNCLEAR | N/A | UNCLEAR |
| Are participants, and their voices, adequately represented? | N/A | N/A | N/A | N/A | YES |
| Is the research ethical according to current criteria or, for recent studies, and is there evidence of ethical approval by an appropriate body? | YES | N/A | YES | N/A | YES |
| Do the conclusions drawn in the research report flow from the analysis, or interpretation, of the data? | YES | YES | YES | YES | YES |


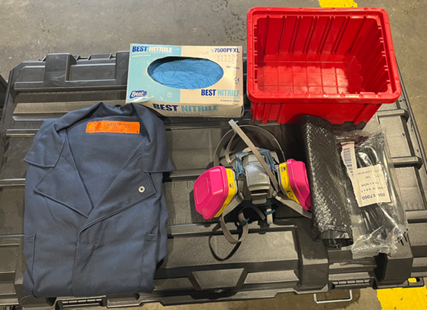


**Supplementary Figure 1:** Required Items
